# Analysis of anticancer compound, indole-3-carbinol, in broccoli using a new ultrasound-assisted dispersive-filter extraction method based on poly(deep eutectic solvent)-graphene oxide nanocomposite

**DOI:** 10.1016/j.jpha.2021.03.013

**Published:** 2021-04-07

**Authors:** Yanan Yuan, Huanhuan Chen, Yehong Han, Fengxia Qiao, Hongyuan Yan

**Affiliations:** a Key Laboratory of Medicinal Chemistry and Molecular Diagnosis of Ministry of Education, College of Pharmaceutical Science, Hebei University, Baoding, Hebei, 071002, China; b Key Laboratory of Public Health Safety of Hebei Province, Institute of Life Science and Green Development, College of Public Health, Hebei University, Baoding, Hebei, 071002, China; c College of Biochemistry, Baoding University, Baoding, Hebei, 071000, China

**Keywords:** Indole-3-carbinol, Broccoli, Poly(deep eutectic solvent)-graphene oxide, Ultrasound-assisted dispersive-filter extraction, Rapid analysis

## Abstract

Indole-3-carbinol (I3C), an important anticancer compound found in broccoli, has attracted considerable attention. The rapid extraction and accurate analysis of I3C in the pharmaceutical industry in broccoli is challenging as I3C is unstable at low pH and high temperature. In this study, a rapid, accurate, and low-cost ultrasound-assisted dispersive-filter extraction (UADFE) technique based on poly(deep eutectic solvent)-graphene oxide (PDES-GO) adsorbent was developed for the isolation and analysis of I3C in broccoli for the first time. PDES-GO with multiple adsorption interactions and a fast mass transfer rate was synthesized to accelerate adsorption and desorption. UADFE was developed by combining dispersive solid-phase extraction (DSPE) and filter solid-phase extraction (FSPE) to realize rapid extraction and separation. Based on the above two strategies, the proposed PDES-GO-UADFE method coupled with high-performance liquid chromatography (HPLC) allowed the rapid (15–16 min), accurate (84.3%–96.4%), and low-cost (adsorbent: 3.00 mg) analysis of I3C in broccoli and was superior to solid-phase extraction, DSPE, and FSPE methods. The proposed method showed remarkable linearity (*r*=0.9998; range: 0.0840–48.0 μg/g), low limit of quantification (0.0840 μg/g), and high precision (relative standard deviation ≤5.6%). Therefore, the PDES-GO-UADFE-HPLC method shows significant potential in the field of pharmaceutical analysis for the separation and analysis of anti-cancer compounds in complex plant samples.

## Highlights

• PDES-GO with multiple adsorption interactions and fast mass transfer was prepared.
• A rapid, accurate, and low-cost sample preparation method-UADFE was developed.
• A new HPLC method was developed based on PDES-GO adsorbent and UADFE.
• The method was applied to accurately determine anticancer substance I3C in broccoli.
• The proposed method shows an application potential in pharmaceutical analysis.

## Graphical abstract

**Figure:**
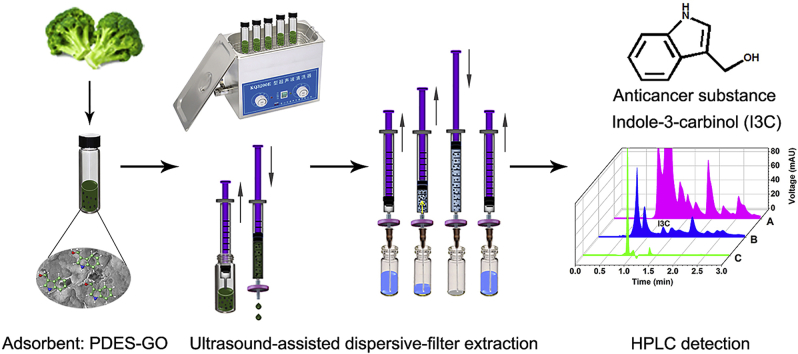

## 1 Introduction

Indole-3-carbinol (I3C) has received significant attention in the field of natural product chemistry owing to its anticancer properties [1,2]. I3C is naturally found in cruciferous plants, particularly broccoli [3]. When the broccoli tissues are damaged (such as during mastication or homogenization), glucobrassicin, an important glucosinolate, comes in contact with myrosinase, an endogenous enzyme present in cruciferous plants, to release I3C [4]. I3C can interact with the WW domain containing E3 ubiquitin protein ligase 1, which affects the function of the tumor suppressor gene phosphatase and tensin homologue to afford anticancer effects [5]. I3C performs anticancer activities against lung, nasopharyngeal, liver, ovarian, and other types of cancer [6,7]. Thus, it is important to develop an analytical method to extract and analyze I3C in broccoli.

It has been reported that I3C is unstable under many conditions such as low pH and high temperature. Under acidic conditions, I3C is rapidly converted into a series of acid condensation compounds [[8], [9], [10]]. The instability of I3C limits its accurate analysis in broccoli. Notably, the sample preparation process accounts for 50%–70% of the entire analysis time [11]. In addition to considering the experimental conditions that are likely to cause I3C instability during its analysis, decreasing the time for sample preparation can effectively reduce the effect of I3C instability on the analytical results; therefore, developing a rapid sample preparation method is necessary.

Most sample preparation methods such as solid-phase extraction (SPE) and derivatization methods rely primarily on the extraction method and the adsorbent [[12], [13], [14], [15]]. Therefore, the rapid extraction and accurate determination of I3C in broccoli can be achieved considering these two aspects [16,17]. Several extraction methods such as SPE, dispersive solid-phase extraction (DSPE), and matrix solid-phase dispersion (MSPD) have been developed [18,19]. SPE affords high recovery and remarkable reproducibility, but the adsorbent amount required is large (tens to hundreds of milligrams), and it often requires an activation step (pretreatment with methanol and water) before loading the sample solution [20,21]. In DSPE, the adsorbent is uniformly dispersed to ensure sufficient contact with the sample solution, washing solvent, and elution solvent, such that adsorption and desorption occur rapidly [22,23]. However, each step in DSPE requires additional centrifugation, which limits the sample preparation process [24]. In MSPD, extraction, filtration, and purification steps are completed in one step, but large amounts of the sample and adsorbent are lost during the transfer of the ground semidry mixture from the mortar to the SPE cartridge [25]. Therefore, a new, rapid, and low-cost extraction method with a low sample loss is required to increase the speed and decrease the cost of the sample preparation method, which meets the requirements for I3C analysis in broccoli.

In addition to the extraction method, adsorbents with high mass transfer rates and high adsorption capacities can accelerate the adsorption and desorption, thereby decreasing the sample preparation time [26,27]. Conventional adsorbents such as C_8_, C_18_, silica, and Al_2_O_3_ cannot meet the requirements of sample preparation because of a single adsorption interaction, low mass transfer rate, and low adsorption capacity. Therefore, several studies have focused on developing new adsorbents. Graphene oxide (GO), which has a two-dimensional sheet structure and a large specific surface area, contains many oxygen-containing functional groups that can be easily modified with other functional groups to improve its adsorption capacity [28,29]. A deep eutectic solvent (DES) is a new type of ionic liquid that can be easily designed and synthesized and has multiple functional groups [[30], [31], [32]]. DES has been used to modify various materials to increase the adsorption interactions and improve the adsorption capacity of the material [[33], [34], [35]]. Therefore, it is proposed that grafting a thin layer of poly(deep eutectic solvent) (PDES) on the GO surface can not only enrich the adsorption interactions and increase the capacity for adsorbing the target analyte, but also afford a high mass transfer rate because of the thin PDES layer, which can increase the speed and extraction efficiency of the sample preparation process for the extraction and analysis of I3C in broccoli.

In this study, a new ultrasound-assisted dispersive-filter extraction (UADFE) method based on the PDES-GO adsorbent was proposed for the rapid, accurate, and low-cost analysis of I3C in broccoli for the first time. The PDES-GO-UADFE method combines the advantages of PDES-GO (high mass transfer rate and high adsorption capacity) and UADFE (rapid extraction and separation). The extraction parameters were optimized, and the proposed method was validated in terms of linearity, accuracy, and precision. Finally, the developed method was applied to analyze I3C in different varieties and parts of the broccoli samples, and it was also used to determine the effect of high-temperature processing on the I3C content in broccoli.

## 2 Experimental

### 2.1 Chemicals and reagents

Choline chloride was purchased from Guangfu Chemical Co., Ltd. (Tianjin, China). Methacrylic acid was purchased from Sigma Aldrich (Shanghai, China). Acetonitrile, methanol, ammonia water (25%), and dichloromethane were purchased from Kermel Chemical Co., Ltd. (Tianjin, China). Silica, NH_2_, strong cation exchange adsorbent, and C_18_ were purchased from Bonna-Agela Technologies (Tianjin, China). Reversible addition-fragmentation chain transfer agent functionalized graphene oxide (GO-RAFT) was synthesized in this laboratory [36]. Standard stock solution (1.00 mg/mL) of I3C was prepared in methanol.

### 2.2 Synthesis and characterization of adsorbent

Choline chloride and methacrylic acid were mixed in a 1:2 M ratio, and the mixture was stirred in a water bath maintained at 80 °C until a transparent and uniform solution (DES) was formed, which was used for the subsequent synthesis of PDES-GO. GO-RAFT (300 mg) was dispersed in acetonitrile (60 mL) and sonicated for 1 h. DES (3.00 g) and 2,2′-azobis(2-methylpropionitrile) (75.0 mg) were then added to the mixture and reacted at 60 °C for 24 h under N_2_ atmosphere to prepare PDES-GO, which was then washed with methanol and water. Thereafter, the material was freeze-dried under vacuum.

The I3C adsorption performance of PDES-GO was evaluated via a static adsorption experiment. After the addition of PDES-GO (2.00 mg) and different concentrations of standard solution (10.0, 30.0, 50.0, 80.0, 100, and 200 μg/mL, 2.0 mL), the centrifuge tubes were placed in a constant temperature oscillator (300 r/min, 25 °C) for 12 h. The supernatant was then analyzed using high-performance liquid chromatography (HPLC).

### 2.3 Sample preparation

Broccoli samples were purchased from the local markets (Baoding, China). For the sample preparation, 100 g of broccoli and 100 g of phosphate buffer solution (PBS, pH 7.4, 0.1 M) were mixed and homogenized for 5 min. Then, 25.0 g of the homogenized mixture was weighed and placed in a constant temperature oscillator at 25 °C for 3 h to release I3C. Thereafter, dichloromethane (10 mL) was added to the mixture, followed by magnetic stirring for 10 min. The mixture was centrifuged (10,000 r/min, 5 min), and the upper solvent layer was transferred into an eggplant-shaped bottle. The extraction process was repeated twice. Subsequently, the organic phase (dichloromethane) was evaporated to dryness (30 °C) and then reconstituted in 5.0 mL of PBS with ultrasonication (thrice). Finally, the reconstituted solution was filtered and transferred into a volumetric flask (25 mL) and diluted up to the mark with PBS.

**Fig. 1:**
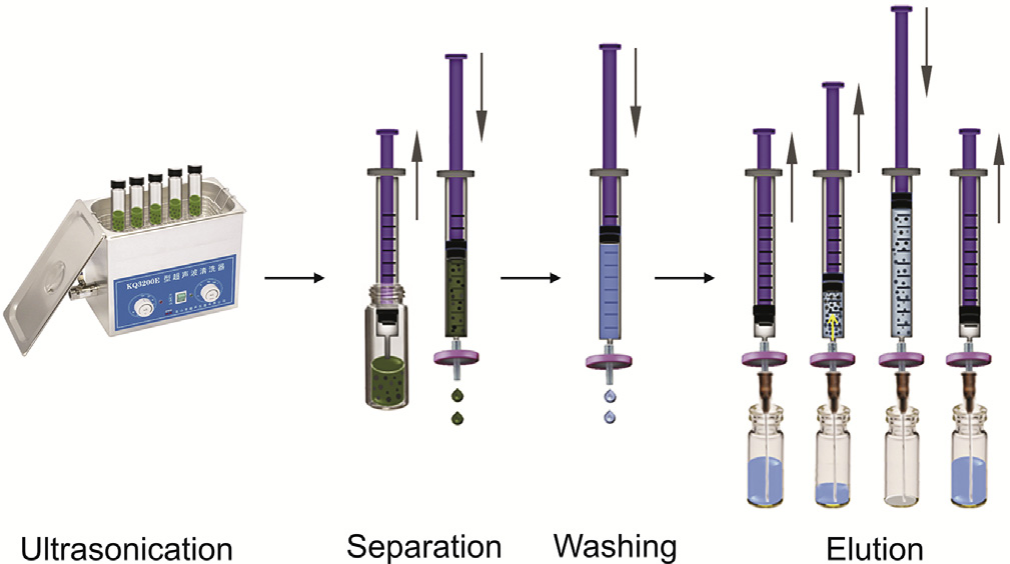
Schematic of poly(deep eutectic solvent)-graphene oxide (PDES-GO)-ultrasound-assisted dispersive-filter extraction (UADFE)-high-performance liquid chromatography (HPLC) method.

The procedure of the PDES-GO-UADFE method is shown in Fig. 1. PDES-GO (3.00 mg) and the sample solution (0.5 mL) were mixed and sonicated for 10 min to extract I3C. The mixture was then drawn into a syringe, and a syringe filter (0.45 μm) was connected to rapidly separate the adsorbent and the sample solution. The syringe filter was unplugged, and 0.5 mL of the washing solvent (PBS) was drawn into the syringe. The same syringe filter was connected to the syringe to wash out the impurities on the adsorbent (~1 min). Thereafter, 1.0 mL of the elution solvent (ammonia water-acetonitrile, 1:9, *V/V*) was drawn into the syringe, and the syringe filter and the needle were connected. Subsequently, the elution solvent was repeatedly drawn up and down into the syringe three times to elute sufficient I3C from the adsorbent (~2 min). This elution process was repeated once with the elution solvent (0.5 mL; ~2 min). Finally, the eluent and PBS were mixed in a 1:1 ratio by vortexing for 10 s, followed by HPLC analysis.

### 2.4 Instruments and analytical conditions

The morphology of PDES-GO was examined by scanning electron microscopy (SEM, Phenom-World BV, Eindhoven, The Netherlands). Fourier-transform infrared (FTIR) spectroscopy was performed using a Thermo Scientific Nicolet iS10 FTIR spectrometer (Thermo Fisher Scientific, Waltham, MA, USA). A Thermo UltiMate3000 DGLC HPLC system (Thermo Fisher Scientific, Waltham, MA, USA) with a Chromeleon 7.2 workstation, diode array detector, and chromatographic column (Accucore C_18_, 100 mm × 4.6 mm, 2.6 μm) was employed for the analysis of I3C. The mobile phase consisted of acetonitrile-water (40:60, *V/V*) at a flow rate of 1.0 mL/min. The analysis wavelength of the detector was 218 nm, and the injection volume was 20 μL.

### 2.5 Comparison of the proposed method with other sample preparation methods

The established extraction method (UADFE) was compared to other sample preparation methods (DSPE, filter solid-phase extraction (FSPE), and commercial SPE) [[37], [38], [39]]. In the extraction process, these sample preparation methods mainly included the following steps: activation, sample loading, washing, and elution. The detailed parameters of the procedure are provided in Table 1 [[37], [38], [39]].

**Table 1:** Procedure of different sample preparation methods.

Method	Adsorbent	Activation	Loading	Washing	Elution	Refs.
FSPE	3.00 mg of PDES-GO	1.0 mL of methanol and PBS	0.5 mL of sample solution	0.5 mL of PBS	1.5 mL of ammonia water-acetonitrile (1:9, *V/V*)	[37]
DSPE	3.00 mg of PDES-GO	Centrifugation for 5 min	0.5 mL of sample solution	0.5 mL of PBS	1.5 mL of ammonia water-acetonitrile (1:9, *V/V*)	[38]
SPE	500 mg of C_18_	1.0 mL of methanol and PBS	0.5 mL of sample solution	0.5 mL of PBS	1.5 mL of ammonia water-acetonitrile (1:9, *V/V*)	[39]
UADFE	3.00 mg of PDES-GO	–	0.5 mL of sample solution	0.5 mL of PBS	1.5 mL of ammonia water-acetonitrile (1:9, *V/V*)	This work

FSPE: filter solid-phase extraction; DSPE: dispersive solid-phase extraction; SPE: solid-phase extraction; UADFE: ultrasound-assisted dispersive-filter extraction; PDES-GO: poly(deep eutectic solvent)-graphene oxide.

## 3 Results and discussion

### 3.1 Synthesis and characterization of adsorbent

**Fig. 2:**
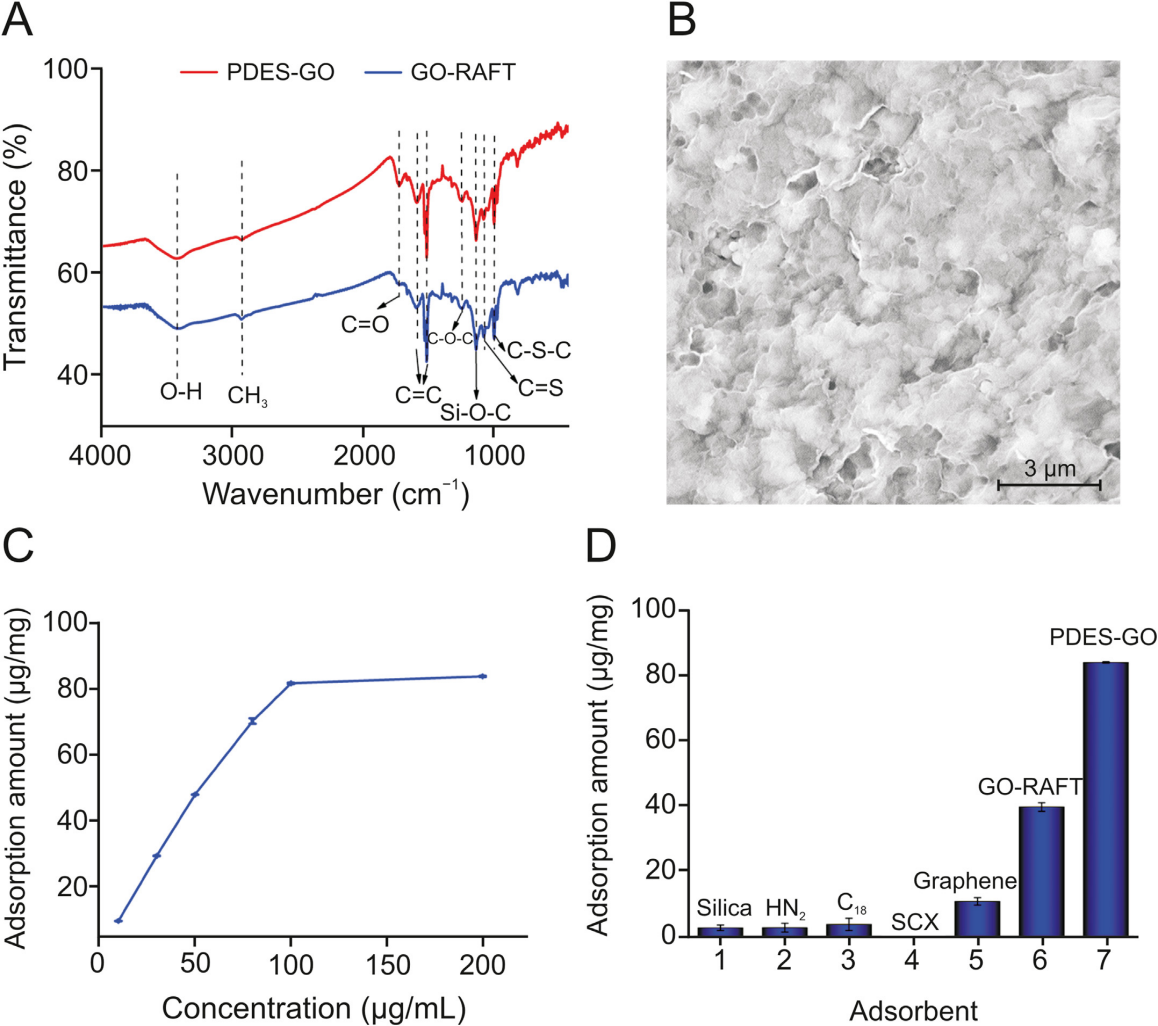
(A) Fourier-transform infrared (FTIR) spectra and (B) scanning electron microscopy (SEM) data of poly(deep eutectic solvent)-graphene oxide (PDES-GO); (C) static I3C adsorption of PDES-GO; (D) I3C adsorption amounts of different adsorbents. GO-RAFT: reversible addition-fragmentation chain transfer agent functionalized GO.

The composition and structure of PDES-GO were determined using FTIR and SEM. As shown in Fig. 2A, the peaks at 1500 and 1600 cm^−1^ correspond to the C=C bonds, which comprise the GO skeleton, and the peaks at 3400, 1715, and 1250 cm^−1^ correspond to the O–H, C=O, and C–O–C bonds of GO, respectively. The peaks at 1100, 1060, and 1000 cm^−1^ were attributed to the Si–O–C, C=S, and –C–S–C– bonds, respectively, indicating that the RAFT agent was grafted onto the GO sheet. The peak intensity of C=O increased in the spectrum of PDES-GO, indicating that GO-RAFT was successfully modified with DES, as DES contains a carboxyl group. In terms of the morphology, the prepared PDES-GO has a three-dimensional skeleton with a porous morphology, based on the SEM data (Fig. 2B). The modification of the RAFT agent and PDES on the GO sheets provides support for GO, such that the PDES-GO sheets are not stacked closely. Around the hole, the edge of the PDES-GO sheets with a thin lamellar structure can be clearly observed, which is conducive to achieving the rapid adsorption and desorption of I3C.

The I3C adsorption performance of PDES-GO was investigated via the static adsorption experiments using a series of I3C standard solutions (10.0–200 μg/mL). As shown in Fig. 2C, a high I3C adsorption with PDES-GO (~80.0 μg/mg) is achieved when the adsorption dynamic equilibrium was attained, which was mainly due to multiple adsorption interactions between the functional groups of PDES-GO and I3C. Thereafter, a series of commercial adsorbents (silica, NH_2_, C_18_, and SCX) with different adsorption interactions were selected to further examine the multiple adsorption interactions of PDES-GO. Silica and NH_2_ can form hydrogen bonding; C_18_ is capable of hydrophobic interactions, and SCX can undergo ion exchange. As shown in Fig. 2D, commercial adsorbents with a single adsorption interaction exhibited poor I3C adsorption performance, which limited their use in the extraction and isolation of I3C. Graphene has a higher adsorption capacity than the commercial adsorbents because graphene is capable of hydrophobic interactions and π–π conjugation for I3C adsorption. PDES-GO exhibits multiple adsorption interactions (π–π conjugation, hydrogen bonding, hydrophobic interactions, and electrostatic adsorption) after modification with PDES and affords the highest I3C adsorption (83.8 μg/mg) among these adsorbents, which demonstrates the excellent adsorption performance of PDES-GO in comparison to the performances of other adsorbents for I3C extraction.

### 3.2 Optimization of sample preparation procedure

As I3C in broccoli does not exist in a prototype form, it is necessary to release I3C through an autolysis step before analysis. In the autolysis step, broccoli was homogenized and placed in a constant temperature oscillator at 25 °C for 3 h [40]. During autolysis, glucobrassicin in broccoli was contacted with myrosinase in cruciferous plants to release I3C. After autolysis, the homogenized mixture was extracted with dichloromethane, followed by rotary evaporation and reconstitution to afford a sample solution containing I3C, which required further extraction and purification. Owing to the instability of I3C, the optimization of a series of extraction parameters (adsorbent amount, ultrasonication time, washing solvent, and elution solvent) in PDES-GO-UADFE was performed to afford rapid extraction and accurate analysis of I3C in broccoli.

#### 3.2.1 Optimization of adsorbent dosage

**Fig. 3:**
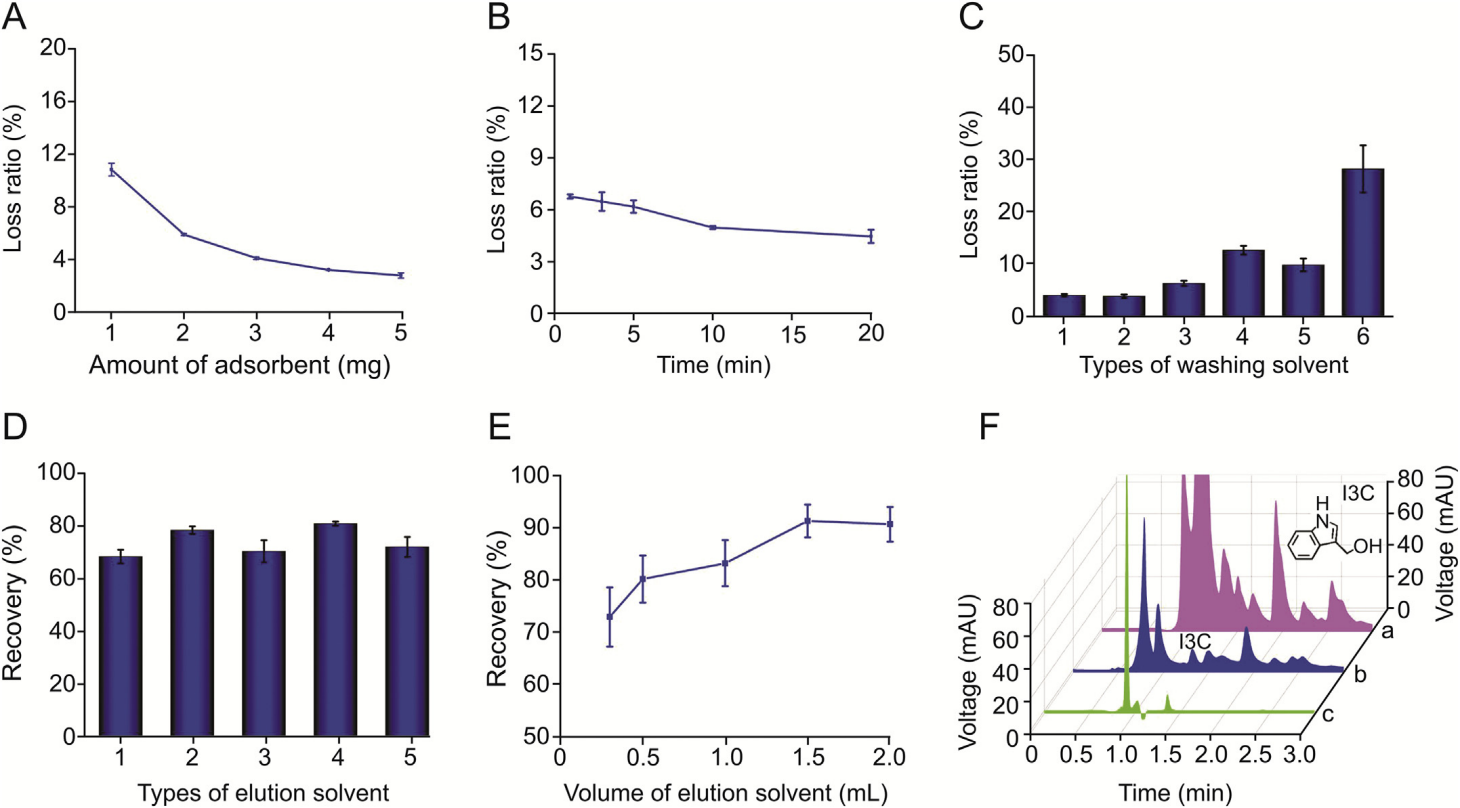
Optimization of (A) adsorbent amount; (B) ultrasonication time; (C) types of washing solvents (1: water; 2: phosphate buffer solution (pH 7.4, 0.1 M); 3: methanol-water (1:9, *V/V*); 4: methanol-water (3:7, *V/V*); 5: acetonitrile-water (1:9, *V/V*); 6: acetonitrile-water (3:7, *V/V*)); (D) types of elution solvents (1: methanol; 2: ammonia water-methanol (1:9, *V/V*); 3: acetonitrile; 4: ammonia water-acetonitrile (1:9, *V/V*); 5: acetonitrile-water (8:2, *V/V*); (E) volumes of ammonia water-acetonitrile (1:9, *V/V*). (F) Chromatograms of broccoli sample before (a) and after (b) pretreatment with PDES-GO-UADFE method, and I3C standard solution (c).

The volume of the sample solution was set to 0.5 mL, and the amount of adsorbent (1.00–5.00 mg) was determined because the adsorption performance of the adsorbent directly affected the extraction efficiency of the analyte (Fig. 3A). When the adsorbent amount was 3.00 mg, the I3C loss ratio was <5%. A small amount of adsorbent was needed mainly because of the multiple interactions of PDES-GO, which could effectively adsorb I3C through π–π conjugation, hydrophobic interactions, electrostatic adsorption, and hydrogen bonding with the functional groups of I3C (benzene ring, –NH–, and –OH).

#### 3.2.2 Optimization of ultrasonication time

As UADFE was a time-dependent extraction procedure, the ultrasonication time for extraction was investigated. As shown in Fig. 3B, suitable extraction results (loss ratio of I3C <5%) were obtained within 10 min of ultrasonication, owing to the thin layer of PDES-GO sheet that facilitated the rapid adsorption and desorption of I3C. Ultrasound-assisted extraction mode facilitated sufficient dispersion and contact of the adsorbent and sample solution for I3C extraction, which improved the extraction efficiency and mass transfer rate of the adsorbent, thereby decreasing the sample preparation time. Therefore, 10 min of ultrasonication was sufficient to afford the rapid extraction of I3C in the sample solution.

#### 3.2.3 Optimization of washing solvent

In the washing step, six types of washing solvents (0.5 mL) were investigated (Fig. 3C). When the washing solvent contained 10%–30% of organic solvent, a high loss of I3C (6.4%–28.2%) occurred. When water or PBS was used as the washing solvent, only a small I3C loss (≤4.1%) was observed. However, water typically dissolves a certain amount of CO_2_, affording a pH of <7, which is not conducive to the stability of I3C; therefore, PBS (0.5 mL) was selected as the washing solvent.

#### 3.2.4 Optimization of elution solvent

A series of elution solvents was then examined to achieve complete I3C elution. As shown in Fig. 3D, the addition of a small amount (10%) of ammonia water to methanol or acetonitrile improved I3C elution because ammonia water can destroy the interactions between I3C and the adsorbent, which include hydrogen bonding and electrostatic adsorption. After the addition of ammonia water, the basic elution solvent was also beneficial for I3C stability. Upon further investigation of the elution solvent volume (Fig. 3E), it was determined that 1.5 mL of ammonia water-acetonitrile (1:9, *V/V*) solution was sufficient to elute I3C from the adsorbent with high recovery (>90%).

### 3.3 Method validation

The PDES-GO-UADFE-HPLC method for I3C analysis was validated by determining the detection limit (LOD), quantification limit (LOQ), linearity, accuracy, and precision. The LOD and LOQ values were 0.0250 and 0.0840 μg/g, respectively, which were determined based on the signal-to-noise (S/N) ratios of 3 and 10, respectively. The calibration curves were constructed by plotting the chromatographic peak area versus a series of analyte concentrations. Sufficient linearity in the range of 0.0840–48.0 μg/g was obtained with a coefficient of determination (*r*) of 0.9998. Accuracy was assessed at three spiking levels of 1.00, 10.0, and 30.0 μg/g (*n*=3), and the recoveries were 84.3%–96.4% with relative standard deviations (RSDs) of ≤5.0%. The method precision was indicated in terms of repeatability and reproducibility, which were calculated by the extraction and analysis of I3C from the broccoli matrix at the spiking level of 10.0 μg/g using the methods in one (intraday, *n*=6) and three consecutive days (interday, *n*=3), respectively. The intraday and interday precisions were expressed as RSDs, which had values ≤5.6%. A wide range (0.0840–48.0 μg/g), good linearity (*r*=0.9998), high sensitivity (LOD: 0.0250 μg/g), high accuracy (84.3%–96.4%), and precision (RSD≤5.6%) of the method showed the application potential of the proposed method for I3C analysis in the broccoli samples.

### 3.4 Analysis of real samples

The developed method was applied to the extraction and analysis of I3C in broccoli. The broccoli solution obtained after autolysis and preliminary solvent extraction contained many interfering substances, which affected the accurate analysis of I3C (Fig. 3F(a)). After pretreatment using the PDES-GO-UADFE method, the interference peaks near I3C disappeared and the I3C peak could be clearly identified (Fig. 3F(b)), indicating the purification effect of the proposed method.

The developed method was applied for I3C analysis in different varieties of broccoli samples, and the results are shown in Table 2. The I3C contents detected in different varieties of broccoli were 6.58 μg/g (sample #1) and 9.11 μg/g (sample #4). The results indicated that broccoli sample #4 had a higher I3C content than broccoli sample #1. This indicates that the proposed method can be used to identify desirable broccoli varieties with high I3C contents.

**Table 2:** Application of the proposed PDES-GO-UADFE-HPLC method for I3C analysis in different varieties of broccoli samples.

Broccoli samples	Part of broccoli	Process	Content (μg/g)
#1	All	No	6.58
#2	Flower bud	No	7.21
#2	Flower stem	No	2.60
#3	Flower bud	No	5.43
#3	Flower stem	No	2.29
#4	All	No	9.11
#4	All	Boiling for 10 min	2.68

Furthermore, the developed method was applied for the analysis of I3C in different edible parts of the broccoli samples. The edible parts of broccoli mainly include the flower bud and the flower stem; the I3C content in these two parts was determined and compared. As shown in Table 2 (samples #2 and #3), the result indicates that the I3C content in the flower bud (5.43–7.21 μg/g) was significantly higher than that in the flower stem (2.29–2.60 μg/g), providing detailed distribution data of I3C in different parts of broccoli.

The developed method was also applied to investigate the effect of high temperature (boiling) on the I3C content in broccoli. The I3C content in broccoli sample #4 was determined before and after processing with boiling water for 10 min, which decreased significantly from 9.11 to 2.68 μg/g. Therefore, it is recommended to reduce the time required for high-temperature processing, which is beneficial for retaining more I3C in broccoli.

### 3.5 Comparison of UADFE with other sample preparation methods

The UADFE method was compared to three common sample preparation methods, namely, FSPE, DSPE, and SPE, to show the advantages of the proposed method. The general operation procedures of FSPE, DSPE, and SPE were based on the literature references [[37], [38], [39]]. In comparison, the types and volumes of solvents used in each step were identical, and only the extraction method was changed for suitable comparison. The results are shown in Table 3. The FSPE procedure was rapid, but the recovery (21.4%–25.2%) was the lowest among the four methods. Owing to the small adsorbent amount (3.00 mg), the adsorbent could not be easily and uniformly spread in the filter; therefore, the adsorbent and the sample solution did not sufficiently contact the FSPE. In DSPE, PDES-GO is hydrophilic and light, which was easily lost upon discarding the sample solution or washing solvent after centrifugation, resulting in a low I3C recovery (51.8%–60.2%). SPE, the most common sample preparation method, had a high recovery of I3C. However, the adsorbent consumption was high (500 mg of C_18_), and the method was more time-consuming (42–52 min) than the other methods. In the UADFE method, the DSPE mode was adopted to afford rapid and sufficient adsorption and desorption, and the FSPE mode was employed to achieve rapid separation of the adsorbent and solution with a high recovery (84.3%–96.4%) and low adsorbent consumption (3.00 mg). Therefore, the UADFE method was better than the other three methods in terms of cost, recovery, and time consumption, indicating that it is more suitable for I3C analysis in the broccoli samples and can meet the requirements of speed, accuracy, and low cost for pharmaceutical analysis.

**Table 3:** Comparison of UADFE method with other sample preparation methods.

Method	Adsorbent (mg)	Time (min)	Recovery (%)	Refs.
FSPE	3.00	12–14	21.4–25.2	[37]
DSPE	3.00	32–34	51.8–60.2	[38]
SPE	500	42–52	100.6–109.7	[39]
UADFE	3.00	15–16	84.3–96.4	This work

## 4 Conclusions

In conclusion, a rapid, accurate, and low-cost PDES-GO-UADFE method was developed for the first time, which was coupled to HPLC for the analysis of I3C in broccoli. In the PDES-GO-UADFE method, PDES-GO with multiple adsorption interactions, high adsorption capacity, and fast mass transfer was synthesized to afford the rapid extraction of I3C, which was superior to that obtained using the commercial adsorbents. The rapid UADFE method with low adsorbent consumption was designed by combining the advantages of DSPE and FSPE to realize rapid adsorption, desorption, and separation. The proposed method was successfully applied to the extraction and analysis of I3C in broccoli, thereby affording a new analytical method for the analysis of anti-cancer compounds in the field of pharmaceutical analysis.

## CRediT author statement

**Yanan Yuan:** Validation, Writing - Original draft preparation; **Huanhuan Chen:** Investigation; **Yehong Han:** Visualization, Methodology; **Fengxia Qiao:** Data curation; **Hongyuan Yan:** Supervision, Writing - Reviewing and Editing.

## Declaration of competing interest

The authors declare that there are no conflicts of interest.

## References

[1] Sarkar F.H., Li Y. (2004). Indole-3-carbinol and prostate cancer. J. Nutr..

[2] Fujioka N., Fritz V., Upadhyaya P. (2016). Research on cruciferous vegetables, indole-3-carbinol, and cancer prevention: a tribute to Lee W. Wattenberg. Mol. Nutr. Food Res..

[3] Lee S.Y., Chu S.M., Lee S.M. (2010). Determination of indole-3-carbinol and indole-3-acetonitrile in *Brassica* vegetables using high-performance liquid chromatography with fluorescence detection. J. Korean. Soc. Appl. Biol. Chem..

[4] Fibigr J., Šatínský D., Havlíková L. (2016). A new method for rapid determination of indole-3-carbinol and its condensation products in nutraceuticals using core-shell column chromatography method. J. Pharm. Biomed. Anal..

[5] Lee Y.-R., Chen M., Lee J.D. (2019). Reactivation of PTEN tumor suppressor for cancer treatment through inhibition of a MYC-WWP1 inhibitory pathway. Science.

[6] Kiselev V.I., Ashrafyan L.A., Muyzhnek E.L. (2018). A new promising way of maintenance therapy in advanced ovarian cancer: a comparative clinical study. BMC Cancer.

[7] Khan N., Mukhtar H. (2015). Dietary agents for prevention and treatment of lung cancer. Cancer Lett..

[8] Anderton M.J., Jukes R., Lamb J.H. (2003). Liquid chromatographic assay for the simultaneous determination of indole-3-carbinol and its acid condensation products in plasma. J. Chromatogr B Analyt. Technol. Biomed. Life Sci..

[9] Hauder J., Winkler S., Bub A. (2011). LC-MS/MS quantification of sulforaphane and indole-3-carbinol metabolites in human plasma and urine after dietary intake of selenium-fortified broccoli. J. Agric. Food Chem..

[10] Li Z., Wei X., Li L. (2017). Development of a simple method for determination of anti-cancer component of indole-3-carbinol in cabbage and broccoli. J. Food Nutr. Res..

[11] Andrade-Eiroa A., Canle M., Leroy-Cancellieri V. (2016). Solid-phase extraction of organic compounds: a critical review (Part I). Trends Anal. Chem..

[12] Dimpe K.M., Nomngongo P.N. (2019). Application of activated carbon-decorated polyacrylonitrile nanofibers as an adsorbent in dispersive solid-phase extraction of fluoroquinolones from wastewater. J. Pharm. Anal..

[13] Jian N., Zhao M., Liang S. (2019). High-throughput and high-efficient micro-solid phase extraction based on sulfonated-polyaniline/polyacrylonitrile nanofiber mats for determination of fluoroquinolones in animal-origin foods. J. Agric. Food Chem..

[14] Jing W., Zhou Y., Wang J. (2019). Dispersive magnetic solid-phase extraction coupled to direct analysis in real time mass spectrometry for high-throughput analysis of trace environmental contaminants. Anal. Chem..

[15] Molaei R., Tajik H., Moradi M. (2019). Magnetic solid phase extraction based on mesoporous silica-coated iron oxide nanoparticles for simultaneous determination of biogenic amines in an Iranian traditional dairy product; Kashk. Food Control.

[16] Chen Y., Xia L., Liang R. (2019). Advanced materials for sample preparation in recent decade. Trends Anal. Chem..

[17] Liu Q., Shi J., Jiang G. (2012). Application of graphene in analytical sample preparation. Trends Anal. Chem..

[18] Seidi S., Tajik M., Baharfar M. (2019). Micro solid-phase extraction (pipette tip and spin column) and thin film solid-phase microextraction: miniaturized concepts for chromatographic analysis. Trends Anal. Chem..

[19] Si R., Han Y., Wu D. (2020). Ionic liquid-organic-functionalized ordered mesoporous silica-integrated dispersive solid-phase extraction for determination of plant growth regulators in fresh *Panax ginseng*. Talanta.

[20] Bai Y.-L., Cai B.-D., Luo X.-T. (2018). Simultaneous determination of abscisic acid and its catabolites by hydrophilic solid-phase extraction combined with ultra high performance liquid chromatography-tandem mass spectrometry. J. Agric. Food Chem..

[21] Yu Q.-W., Sun H., Wang K. (2017). Monitoring of carbendazim and thiabendazole in fruits and vegetables by SiO_2_@NiO-based solid-phase extraction coupled to high-performance liquid chromatography-fluorescence detector. Food Anal. Methods.

[22] Custodio-Mendoza J.A., Lorenzo R.A., Valente I.M. (2018). Development of a partitioned liquid-liquid extraction-dispersive solid phase extraction procedure followed by liquid chromatography-tandem mass spectrometry for analysis of 3-monochloropropane-1,2-diol diesters in edible oils. J. Chromatogr. A.

[23] Mao X., Yan A., Wan Y. (2019). Dispersive solid-phase extraction using microporous sorbent UiO-66 coupled to gas chromatography-tandem mass spectrometry: a QuEChERS-type method for the determination of organophosphorus pesticide residues in edible vegetable oils without matrix interference. J. Agric. Food Chem..

[24] Panjan P., Monasterio R.P., Carrasco-Pancorbo A. (2018). Development of a folic acid molecularly imprinted polymer and its evaluation as a sorbent for dispersive solid-phase extraction by liquid chromatography coupled to mass spectrometry. J. Chromatogr. A.

[25] Li J., Li Y., Xu D. (2017). Determination of metrafenone in vegetables by matrix solid-phase dispersion and HPLC-UV method. Food Chem..

[26] Kong D., Qiao N., Liu H. (2017). Fast and efficient removal of copper using sandwich-like graphene oxide composite imprinted materials. Chem. Eng. J..

[27] Ning F., Qiu T., Wang Q. (2017). Dummy-surface molecularly imprinted polymers on magnetic graphene oxide for rapid and selective quantification of acrylamide in heat-processed (including fried) foods. Food Chem..

[28] Wu J., Liang X., Hao L. (2017). Graphene oxide cross-linked with phytic acid: an efficient adsorbent for the extraction of carbamates. Microchim. Acta.

[29] Tursynbolat S., Bakytkarim Y., Huang J. (2019). Highly sensitive simultaneous electrochemical determination of myricetin and rutin via solid phase extraction on a ternary Pt@r-GO@MWCNTs nanocomposite. J. Pharm. Anal..

[30] Farajzadeh M.A., Abbaspour M., Kazemian R. (2020). Preparation of a new three-component deep eutectic solvent and its use as an extraction solvent in dispersive liquid-liquid microextraction of pesticides in green tea and herbal distillates. J. Sci. Food Agric..

[31] Cunha S.C., Fernandes J.O. (2018). Extraction techniques with deep eutectic solvents. Trends Anal. Chem..

[32] Ruesgas-Ramón M., Figueroa-Espinoza M.C., Durand E. (2017). Application of deep eutectic solvents (DES) for phenolic compounds extraction: overview, challenges, and opportunities. J. Agric. Food Chem..

[33] Huang Y., Wang Y., Pan Q. (2015). Magnetic graphene oxide modified with choline chloride-based deep eutectic solvent for the solid-phase extraction of protein. Anal. Chim. Acta.

[34] Li X., Row K.H. (2017). Purification of antibiotics from the millet extract using hybrid molecularly imprinted polymers based on deep eutectic solvents. RSC Adv..

[35] Yousefi S.M., Shemirani F., Ghorbanian S.A. (2017). Deep eutectic solvent magnetic bucky gels in developing dispersive solid phase extraction: application for ultra trace analysis of organochlorine pesticides by GC-micro ECD using a large-volume injection technique. Talanta.

[36] Yuan Y., Nie H., Yin J. (2020). Selective extraction and detection of β-agonists in swine urine for monitoring illegal use in livestock breeding. Food Chem..

[37] Han Y., Yang C., Zhou Y. (2017). Ionic liquid-hybrid molecularly imprinted material-filter solid-phase extraction coupled with HPLC for determination of 6-benzyladenine and 4-chlorophenoxyacetic acid in bean sprouts. J. Agric. Food Chem..

[38] Wang C., Li X., Yu F. (2021). Multi-class analysis of veterinary drugs in eggs using dispersive-solid phase extraction and ultra-high performance liquid chromatography-tandem mass spectrometry. Food Chem..

[39] Pilipczuk T., Dawidowska N., Kusznierewicz B. (2015). Simultaneous determination of indolic compounds in plant extracts by solid-phase extraction and high-performance liquid chromatography with UV and fluorescence detection. Food Anal. Methods.

[40] Kokotou M.G., Revelou P.-K., Pappas C. (2017). High resolution mass spectrometry studies of sulforaphane and indole-3-carbinol in broccoli. Food Chem..

